# A Digital Health Intervention Platform (Active and Independent Management System) to Enhance the Rehabilitation Experience for Orthopedic Joint Replacement Patients: Usability Evaluation Study

**DOI:** 10.2196/50430

**Published:** 2024-05-14

**Authors:** Petros Papadopoulos, Mario Soflano, Thomas Connolly

**Affiliations:** 1 University of Strathclyde Glasgow United Kingdom; 2 Glasgow Caledonian University Glasgow United Kingdom; 3 DS Partnership Glasgow United Kingdom

**Keywords:** mobile health, mHealth, digital health intervention, total knee replacement, TKR, total hip replacement, THR, dynamic hip screw, DHS, rehabilitation, usability, mobile phone

## Abstract

**Background:**

Optimal rehabilitation programs for orthopedic joint replacement patients ensure faster return to function, earlier discharge from hospital, and improved patient satisfaction. Digital health interventions show promise as a supporting tool for re-enablement.

**Objective:**

The main goal of this mixed methods study was to examine the usability of the AIMS platform from the perspectives of both patients and clinicians. The aim of this study was to evaluate a re-enablement platform that we have developed that uses a holistic systems approach to address the *de-enablement* that occurs in hospitalized inpatients, with the older adult population most at risk. The Active and Independent Management System (AIMS) platform is anticipated to deliver improved patient participation in recovery and self-management through education and the ability to track rehabilitation progression in hospital and after patient discharge.

**Methods:**

Two well-known instruments were used to measure usability: the System Usability Scale (SUS) with 10 items and, for finer granularity, the User Experience Questionnaire (UEQ) with 26 items. In all, 26 physiotherapists and health care professionals evaluated the AIMS clinical portal; and 44 patients in hospital for total knee replacement, total hip replacement, or dynamic hip screw implant evaluated the AIMS app.

**Results:**

For the AIMS clinical portal, the mean SUS score obtained was 82.88 (SD 13.07, median 86.25), which would be considered *good/excellent* according to a validated adjective rating scale. For the UEQ, the means of the normalized scores (range −3 to +3) were as follows: attractiveness=2.683 (SD 0.100), perspicuity=2.775 (SD 0.150), efficiency=2.775 (SD 0.130), dependability=2.300 (SD 0.080), stimulation=1.950 (SD 0.120), and novelty=1.625 (SD 0.090). All dimensions were thus classed as *excellent* against the benchmarks, confirming the results from the SUS questionnaire. For the AIMS app, the mean SUS score obtained was 74.41 (SD 10.26), with a median of 77.50, which would be considered *good* according to the aforementioned adjective rating scale. For the UEQ, the means of the normalized scores were as follows: attractiveness=2.733 (SD 0.070), perspicuity=2.900 (SD 0.060), efficiency=2.800 (SD 0.090), dependability=2.425 (SD 0.060), stimulation=2.200 (SD 0.010), and novelty=1.450 (0.260). All dimensions were thus classed as *excellent* against the benchmarks (with the exception of novelty, which was classed as *good*), providing slightly better results than the SUS questionnaire.

**Conclusions:**

The study has shown that both the AIMS clinical portal and the AIMS app have *good* to *excellent* usability scores, and the platform provides a solid foundation for the next phase of research, which will involve evaluating the effectiveness of the platform in improving patient outcomes after total knee replacement, total hip replacement, or dynamic hip screw.

## Introduction

### Background

According to the World Health Organization’s 2019 Global Burden of Disease study, approximately 1.71 billion people globally experience musculoskeletal conditions. Low back pain is the most common condition, affecting an estimated 568 million people [1]. In the United Kingdom, it has been estimated that musculoskeletal conditions affect >20 million people, approximately a third of the population [2]. Musculoskeletal conditions are the second greatest contributor to disability worldwide and is a significant burden to the individual and society [3]. It is expected that the impact of musculoskeletal conditions on the health service and on society will continue to rise as life expectancy increases [4]. Many different approaches have been explored to reduce this burden, including medical interventions, work-related approaches (reducing stress at work as well as improving health and safety regulations), social education (improving awareness of exercise and healthy eating), and the use of technology.

Musculoskeletal conditions comprise >150 different disorders, diseases, and syndromes that affect bones, joints, muscles, the spine, and soft tissues [3]. While some conditions are short lived, such as sprains and fractures, others can be lifelong conditions requiring ongoing treatment. Pain is a common symptom of musculoskeletal conditions. Back and neck pain, osteoarthritis, rheumatoid arthritis, and fractures are among the most disabling conditions and can be a significant barrier to healthy aging [5]. Musculoskeletal conditions can be classified by the body part affected (eg, knee pain and shoulder pain), whether the condition is noninflammatory (such as osteoarthritis) or inflammatory (such as rheumatoid arthritis), and whether the condition is restricted to the musculoskeletal system or more widespread (such as systemic lupus erythematosus) [4]. To compound matters, musculoskeletal issues tend to be associated with other diseases, such as heart or respiratory disease and stroke, and lead to an increase in disabilities and deaths [6-8]. It has been estimated that musculoskeletal conditions account for up to 21% of annual general practitioner consultations across England [9], and health service costs from inability to work and sickness absence in the United Kingdom are approximately £100 billion (US $125 billion) annually [10]. It is important to find solutions that will help reduce the significant burdens on the individual, society, the economy, and the health service. While many solutions will be of a medical nature, technology has a significant part to play in easing the burdens. In the next subsection, we discuss some digital health interventions (DHIs) for musculoskeletal conditions.

A number of different terminologies exist in the health domain for software solutions generally. The terms eHealth and mobile health (mHealth) have been used for a number of years. More recently, the more encompassing term *digital health* has been introduced. This is defined as “encompassing eHealth [which includes mHealth] as well as developing areas such as the use of advanced computing sciences (in the fields of ‘big data,’ genomics and artificial intelligence, for example)” [11]. Examples of digital health solutions include primary and secondary care IT systems; patient portals that provide secure web-based access to a range of health services, such as My Diabetes My Way and PatientView [12]; personal health data stores such as Mydex [13]; telehealth systems such as Attend Anywhere and Near Me [14]; and health-related mobile apps. It is believed that these systems can benefit health care delivery by improving different outcomes, such as effectiveness, efficiency, accessibility, safety, and personalization [15]. There has been a growing public interest in DHIs because they can allow individuals to monitor, manage, and improve their health and quality of life in a more personalized way, potentially more cost-effectively, and at a time that suits them [16-18].

Optimal rehabilitation programs for orthopedic joint replacement patients ensure faster return to function, earlier discharge from hospital, and improved patient satisfaction [4,19-21] as well as prevent further deconditioning [22]. The aim of this study was to evaluate the usability of a re-enablement platform called Active and Independent Management System (AIMS) that was developed to address the *de-enablement* that occurs in hospitalized inpatients for one of the groups considered to be most at risk, that is, older adults. The platform is capable of delivering digital rehabilitation plans and tracking the progression of the plans in real time; in addition, it can be used both in hospital and at home after a patient is discharged. The rationale for using such a system is to help reduce the time spent in hospital and improve patient satisfaction through self-management.

### Re-Enablement DHIs

This subsection examines some recent literature related to the use of DHIs for total knee replacement (TKR) or total knee arthroplasty (TKA) and total hip replacement (THR) or total hip arthroplasty (THA). Hussain et al [23] developed a TKR platform comprising a mobile phone app, a wrist-worn activity tracker, and a clinical web portal. The purpose-built iOS and Android apps included weekly psychoeducation sessions and tasks that were delivered by a program guide via text and voice recordings. By obtaining the data from the tracker and the app, the clinician could monitor patient progress and the configured physiotherapy programs, while the patient care team could review the progress and the designated programs using the web portal. Physiotherapy programs were mostly from a library of videos created for TKR rehabilitation, which were made available in the app once set by the clinician. The authors planned to conduct a 13-month multisite unblinded randomized controlled trial in which participants were assigned to 1 of 2 study groups [23]. The participants for the experiment were patients who underwent TKR, and the study included an active intervention period from the time the patients were scheduled for surgery (approximately 4 weeks before surgery) to 12 weeks after the surgery, followed by a 40-week free-living period until 1 year after surgery.

Timmers et al [24] investigated the effect of a mobile app for day-to-day postoperative care education on TKR patients regarding the level of pain compared to those who only received standard information about their recovery through the app. The study involved 114 patients in the intervention group and 99 patients in the control group. In the intervention group, 93 patients downloaded and used the app. The results showed that, in comparison with standard patient education, the active education and coaching of patients on a day-to-day basis via the app in the 4 weeks after TKR resulted in a significant decrease, among other things, in the patients’ levels of pain and a significant improvement in patients’ physical functioning and quality of life, as well as their ability to perform physiotherapy exercises and activities of daily self-care.

Van Dijk-Huisman et al [25] developed a mobile app to prevent the negative effects of inactivity in hospital. The app supported objective activity monitoring, gave patients a view of their recovery progress, and offered a customized exercise program. The aim of the study was to investigate the potential of the app to enhance physical activity levels and functional recovery after orthopedic surgery discharge. In all, 97 patients undergoing TKA and THA were recruited for the evaluation. The control group (n=64) received standard physiotherapy, while the intervention group (n=33) used the mobile app in addition to physiotherapy. The time spent in active and functional recovery on postoperative day 1 (POD1) was measured. The app use, corrected for age, resulted in patients standing and walking on POD1 for an average increase of 28.43 (95% CI 5.55-51.32) minutes. The odds of achieving functional recovery on POD1 were 3.08 times higher (95% CI 1.14-8.31) with the use of the mobile app. The authors concluded that a mobile app combined with an accelerometer demonstrated the potential to enhance patients’ activity levels and functional recovery during their hospital stay [25].

Wijnen et al [26] investigated the effectiveness of a home-based rehabilitation program using a tablet app and remote coaching for patients after THA. Existing data from 2 studies were combined: patients from a single-arm intervention study were matched with the historical controls from an observational study. Patients aged 18 to 65 years who had undergone THA were included. The intervention group had a 12-week home-based rehabilitation program with instructional videos on a tablet device and remote coaching. Patients were asked to perform strengthening and walking exercises at least 5 days a week. The intervention group was compared with a control group that included patients who received usual care. Effectiveness was measured at 4 points (preoperatively and 4 weeks, 12 weeks, and 6 months postoperatively) by means of functional tests and self-reported questionnaires. The intervention group performed functional tests significantly faster at 12 weeks and 6 months postoperatively and also scored significantly higher on the subscales *function in sport and recreational activities* and *hip-related quality of life* of the Hip Disability and Osteoarthritis Outcome Questionnaire, as well as on the subscale *physical role limitations* of the Short Form Health Survey-36 at 12 weeks and 6 months postoperatively. Large effect sizes were found on functional tests at 12 weeks and 6 months, endorsed by effect sizes on the self-reported outcomes. The authors concluded that the results demonstrated larger effects in the intervention group than in the historical controls, indicating that a home-based rehabilitation program using a mobile app after THA can be more effective than usual care [26].

Bell et al [27] ran a controlled pilot study for TKR patients, investigating the feasibility and effectiveness of interACTION, a remote (wearable) rehabilitation monitoring platform developed for use by patients after TKR. The InterACTION platform has portable motion sensors placed on either side of a joint to collect joint orientation data using a custom mobile app and then send the data to the clinician’s web-based portal. The mobile app also contains 30 knee-specific home exercises for TKR rehabilitation that the physical therapist can personalize remotely through a web-based clinical portal. The study compared 2 groups: 19 patients who used the interACTION platform and a control group with 19 patients who used standard postoperative outpatient rehabilitation with a physical therapist (2-3 sessions per week over a maximum of 10 weeks), supplemented with a home exercise program. The primary outcome measured was value, operationally defined as the change in the activities of daily living scale of the Knee Outcome Survey at 10 weeks divided by the total cost of rehabilitation (determined from the total number of physical therapy sessions and the billable charges for each session during the 10 weeks the patients were enrolled in the study). In terms of this measure, no statistical differences were found between the groups. The study showed relatively low and not significant differences between the groups in terms of attrition rates, indicating that both interventions were acceptable. There was a small decrease in clinic visits by patients in the interACTION group, and all patients and physical therapists in the group indicated that they would use the system again.

Bäcker et al [28] developed a mobile app with a GenuSport sensor that allows isokinetic exercises to improve postoperative quadriceps weakness and knee motion. The sensor was placed underneath the patient’s knee, and gamified exercise routines were presented through the app consisting of two exercises: (1) *high striker game*, where the patient has to push the knee onto the sensor for 5 seconds; and (2) *flight simulator*, where the player is supposed to keep the knee in the air for 100 seconds. The authors carried out a randomized controlled trial with a 2-year follow-up to evaluate the effectiveness of the app-based rehabilitation for patients after TKA [28]. In all, 35 patients completed the study and were randomly assigned to 2 groups: 20 patients received the app-based exercise program, and 15 patients were included in the control group. Patients in the app group used an external device to measure knee range of motion starting on the day of surgery, whereas patients in the control group underwent regular physiotherapy. Functional outcome scores using the Knee Injury and Osteoarthritis Outcome Score, the Knee Society Scoring System, and a visual analog scale for pain were analyzed. The results showed that, in the short term, the app group performed significantly better than the control group when taking a 10-minute walk, with less pain. In the longer term, the app group also performed significantly better, with higher Knee Society Scoring System scores as well as requiring fewer painkillers. In addition, the app group participants were more likely to participate in sports.

Colomina et al [29] developed an mHealth system for older patients with complex chronic conditions undergoing elective THA or TKA. The mHealth system formed part of the Personalized Connected Care for Complex Chronic Patients platform, which contained a web-based smart adaptive case management system for health care professionals that seamlessly integrated with a patient self-management mHealth system that supported communication between health care professionals and patients. The authors assessed the effectiveness and cost-effectiveness of implementing an mHealth-enabled integrated care (IC) model for patients with complex chronic conditions undergoing TKA or THA versus usual care [29]. A prospective pragmatic 2-arm parallel implementation trial was conducted in the rural region of Lleida in Catalonia, Spain, for 3 months. A total of 29 patients with complex chronic conditions undergoing TKA or THA and their caregivers received the IC program, while 30 patients with statistically comparable baseline characteristics, such as age, sex, and type of arthroplasty, were recruited for the usual care group. The results suggested that both treatment models significantly improved the physical and mental health status of the patients; however, IC significantly reduced the number of unplanned visits related to the surgery procedure and consequently significantly lowered the patients’ expenses.

Rian et al [30] presented a web tool called Eir for symptom registration at home after knee arthroplasty. Given that the system was previously used in cancer care, a separate patient module was designed for patient-reported postoperative symptom assessment and medication registration after fast-track TKA that consisted of measurements of pain and side effects, as well as detailed registration of the use of analgesic drugs. The authors conducted a usability and feasibility study using a randomized controlled trial involving 134 participants [30]. The tool’s usability was assessed with the use of the System Usability Scale (SUS) by 119 of the 134 participants, while the feasibility data were collected qualitatively. The results showed that 70% of the participants managed to use the tool at home without any technical support, although they indicated technical challenges related to the log-in procedure or internet access. The usability was rated high, with a mean SUS score of 89.6 (median 92.5; range 22.5-100).

Two literature reviews assessing the use of app-based rehabilitation for TKA or THA were conducted recently [31,32]. Bäcker et al [31] examined the functional outcomes of app-based rehabilitation of patients after TKA or THA. The review identified 420 entries from MEDLINE or PubMed and Google databases, but only 9 publications met the inclusion criteria, covering 518 patients in the intervention groups and 549 patients in the control groups. Five studies used app-based exercise instructions delivered via a mobile device, and 4 studies used a sensor or motion tracker. The average follow-up was 9.5 (SD 8.1; range 3-23.4) months. Overall, significantly lower activity visual analog scale values were observed for the interventional groups in the short term (*P*=.002). There were no other significant differences observed between the 2 groups. The study found that there were significant short-term improvements in the mobile app group. The authors concluded that mobile apps provide an alternative to in-person sessions that may improve access to physical activity for patients after TKA or THA, and, in combination with a Bluetooth-enabled sensor for isometric exercises, patients can additionally receive real-time feedback after TKA or THA [31].

Constantinescu et al [32] conducted a systematic literature review on the use of commercially available smartphone apps and wearable devices to assist rehabilitation interventions after TKA from the PubMed, Cochrane Library, MEDLINE, and Web of Science databases. Of the 60 full-text studies identified (published between January 2020 and September 2021), a total of 15 met the inclusion criteria, of which 4 studies used smartphone apps, 7 used wearable devices, and 4 used both to monitor physical activity and patient status after TKA. In terms of primary outcomes, 3 studies examined device accuracy, 3 recovery prediction, 2 functional recovery, 2 physical activity promotion, 2 patient compliance, 2 pain control, and 1 study examined health care use. The authors concluded that commercially available apps and wearable devices can capably monitor physical activity and improve patient engagement after TKA, making them approaches that support or replace traditional rehabilitation programs [32]. Using different strategies in interventions, such as setting step goals, using app-based patient engagement platforms, and establishing patient-specific benchmarks for recovery, can enhance the effectiveness of the treatment.

### The AIMS Platform

It is well established that musculoskeletal conditions contribute to a large number of disabilities worldwide, and the projections show that this number will continue to rise. Initiatives are ongoing to combat this problem proactively (eg, reducing stress, improving health and safety regulations, exercising, and healthy eating). Furthermore, reactive approaches of optimal prehabilitation and rehabilitation programs are also undergoing development to optimize operations and ensure the best use of available resources while improving patient satisfaction. The aim of this study was to evaluate a rehabilitation platform in an effort to combat the lack of enablement in hospitalized older adults considered more vulnerable. The platform is capable of delivering digital rehabilitation plans and tracking the progression of these plans in real time; in addition, it can be used both in hospital and at home after patient discharge. The rationale for using such a system is to help reduce time spent in hospital and improve patient satisfaction through self-management.

**Figure 1:**
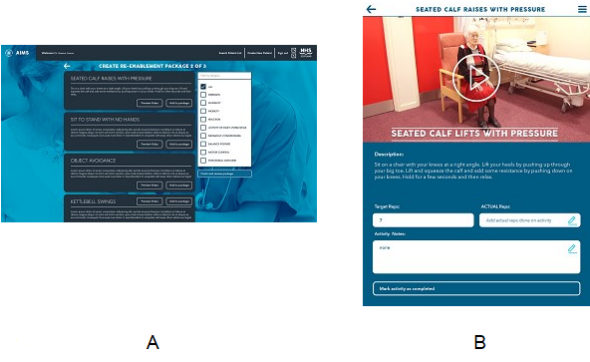
(A) The Create Rehabilitation Re-Enablement Package Screen, and (B) the patient exercise screen.

The AIMS platform helps manage patients’ rehabilitation programs. Each patient is registered by a clinician at the beginning of their patient pathway, and the system collects certain relevant information about the patient as they move through their journey. Rehabilitation clinicians use this platform to create, monitor, and adjust a patient’s rehabilitation package as and when required. A team consisting of stakeholders is assigned to each patient and is responsible for the delivery of the program. A library of physiotherapy exercises and educational videos (eg, the use of a walking aid or how to apply a patient’s splint) have been recorded and uploaded into the system (Figure 1). Each staff member can attach a series of video exercises specific to the patient’s needs that can help the re-enablement process.

The platform consists of 2 components: a web content management system used by clinicians to create rehabilitation plans for postoperative patients and a mobile app designed to deliver these rehabilitation plans to the patients with a series of exercises to be completed by them.

The clinician starts by creating a user account for a patient (all patient information is anonymized with a random unique ID number that is later used to gain access to the rehabilitation plan through the app; Figure 1). The clinician then sets up a specific rehabilitation plan for this patient according to their needs. The clinician can search from all available exercises by using general search terms to filter what is available and also preview the associated video to make sure the appropriate plan is created (Figure 1A). The clinician must then determine the number of repetitions for each exercise and the frequency at which they need to be performed each day, usually 4 sessions a day. After the plan is complete, it becomes active on the patient’s device, and the clinician then demonstrates to the patient how to use the app. This enables the clinician to monitor a patient and their progress each time they complete an exercise. There is also capability for the patient to comment on any particular issues with any of the exercises, and the clinician will be able to view the comments using the content management system and modify the plan accordingly. A typical example of a rehabilitation plan for a patient consists of some simple but very effective exercises (eg, heel slides, knee extensions, and knee flexions). The patient would be asked to perform 10 repetitions of all exercises in 4 daily sessions. The rehabilitation process starts after the operation for as long as the patient remains in hospital, and there are physiotherapists available to offer assistance during the patient rehabilitation process; the app does not prompt patients to complete their daily rehabilitation plan because these sessions are already scheduled in the hospital ward. It is up to the patient to continue using the app for rehabilitation after hospital discharge (the app is available for free download from app stores).

The patient uses a tablet device provided by the hospital to gain access to the AIMS mobile app and work on their rehabilitation plan. Each user is given a random ID number generated by the clinician that is required to log in to the app; no password is required because all information is anonymized. After this, the user can use the app and work on their specific rehabilitation plan and set of exercises and also view their daily plan progress. The patient is provided a textual description of the exercise and a video with audio explaining how it should be performed and how many repetitions should be performed (Figure 1B). At the bottom of the page featuring each exercise, feedback can be provided on how many repetitions were achieved as well as any comments if there were any issues when performing the exercise.

Typically, the app would be used by a member of the staff or a member of the family during visiting hours to help the patient with their exercises by encouraging them or participating with them and achieving successful completion of the rehabilitation plan.

### Aims of the Study

The aim of this study was to investigate the usability of the AIMS platform from the perspectives of both clinicians and patients. Two well-known instruments were used to measure usability: the SUS [33] with 10 items and, for finer granularity, the User Experience Questionnaire (UEQ) [34] with 26 items.

The evaluation aims to answer the following 2 research questions (RQs):

- RQ1: does the AIMS clinical portal provide a solution that could be usable by clinicians?
- RQ2: does the AIMS app provide a solution that could be usable by patients?

## Methods

### Overview

The World Health Organization defines evaluation as “the systematic and objective assessment of an ongoing or completed project [with the aim of determining] the relevance and fulfilment of objectives, development efficiency, effectiveness, impact and sustainability” [35]; and the guide for monitoring and evaluating DHIs outlines 7 stages of DHI maturity, ranging from preprototype to full deployment. This project is considered to be at the prototype stage of maturity, which would include usability testing. Ways to improve the system would also be investigated.

Usability is recognized as a significant quality indicator that determines the success of software applications [36-39]. Johnson et al [40] defines three main approaches to evaluate usability: (1) user based (a sample of prospective users use the system), (2) expert based (≥1 usability or human-computer interaction experts evaluate the system), and (3) model based (formal methods are used to predict user performance). Our health board members were keen on using the user-based approach to evaluate the DHI; hence, this approach was chosen.

Many validated usability instruments have been proposed in the literature with varying numbers of questions. In this study, we used 2 well-known validated instruments: the SUS and the UEQ. The SUS [33] consists of 10 statements (5 positive and 5 negative) that the users rate on a scale ranging from 1=*strongly disagree* to 5=*strongly agree*. The questionnaire alternates between positive and negative statements to avoid random answers. The aggregated score out of 100 can be compared with the average SUS benchmark score of 68.0. To represent SUS scores, Bangor et al [41] defined a 7-point adjective rating scale: *best imaginable*, *excellent*, *good*, *OK*, *poor*, *awful*, and *worst imaginable*.

The UEQ assesses the extent to which (1) the product meets expectations and (2) a product can be compared with other systems using a published benchmark. Schrepp et al [42] developed an adjective rating scale for benchmarking, and a mean score of >1.75 would be considered in the 10% best results. While the UEQ provides finer detail than the SUS, it was felt that asking busy clinicians to rate 26 statements may result in a smaller number of responses compared to asking them to rate 10 SUS statements; therefore, it was decided to use the SUS with all participants and the UEQ with a small number of participants.

Some qualitative information was also gathered using open-ended questions to gain a deeper understanding of participants’ views of the AIMS platform.

### Ethical Considerations

Ethics approval for this study was obtained from Hairmyres University Hospital, Lanarkshire. One of the conditions of approval was that all personal information from the study should be removed and that patient information should be kept private and safe (Data Protection Impact Assessment Questionnaire for Active Independent Mobility System [AIMS] Pilot Study Hairmyres University Hospital, Lanarkshire; May 29, 2019). All participants provided consent before participating in the study.

### Participants

In all, 26 physiotherapists and health care professionals volunteered to evaluate the AIMS clinical portal; and 44 patients in hospital for TKR, THR, or dynamic hip screw (DHS) agreed to participate in evaluating the AIMS app. The study was carried out on May 5, 2019, or November 14, 2019.

### Test Protocol

**Figure 2:**
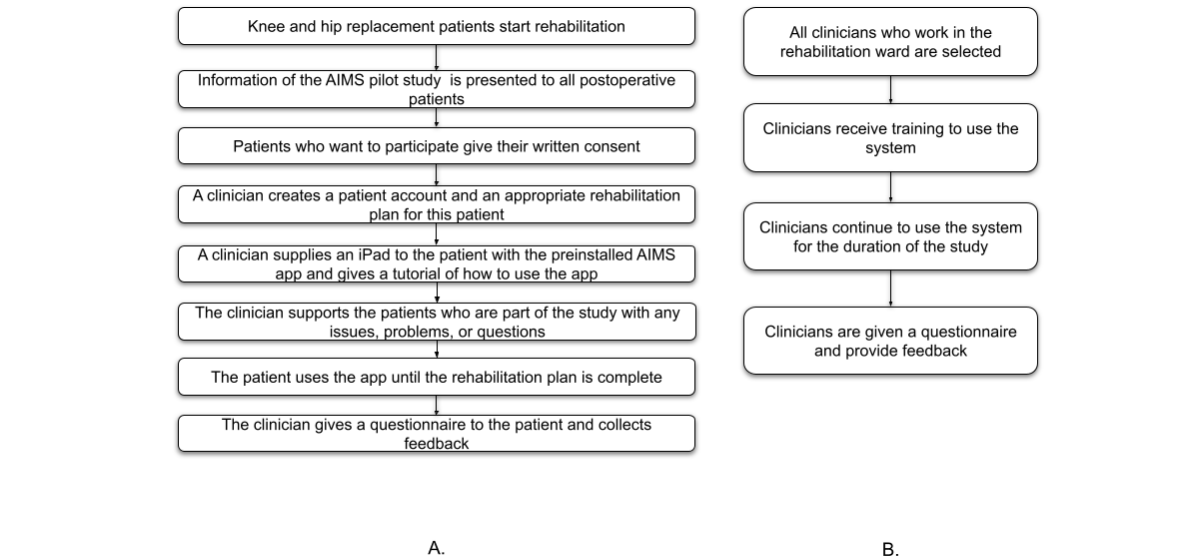
(A) Patient test protocol. (B) Clinician test protocol. AIMS: Active and Independent Management System.

The 6-month-long study was undertaken in the rehabilitation ward in an Hairmyres University Hospital, Lanarkshire, that specializes in TKR or THR surgery. Only clinicians had access to the patients during their stay in the hospital, and a member of the team of clinicians (lead study clinician) had oversight over recruiting and running the experiment. Technical support was provided by the study team to all clinicians during the rehabilitation sessions in the hospital, but this was not in the rehabilitation ward. Questionnaires were given to the participants in a paper-based form, which they were asked to complete and hand back to the lead study clinician toward the end of their rehabilitation stay. The study was conducted using 10 hospital-supplied second-generation iPad Air 2 devices running iOS 10.3 with a 9.7-inch display in portrait orientation (refer to the patient test protocol presented in Figure 2A).

Clinicians of the rehabilitation team in the hospital were all given training on how to use the portal to create rehabilitation packages for patients and how they would look in the app. They were also involved in the development and design process with focus groups and early prototyping, which enabled most of them to develop a good understanding of the AIMS platform. As the study was taking place alongside patients who were not part of the study, everyone had to be able to help the patients, which is why they were all trained to use the system. Clinicians were given a questionnaire to complete after they had used the platform a few times (refer to the clinician test protocol presented in Figure 2B).

## Results

For the SUS, the analysis was carried out using Excel (Microsoft Corp); and for the UEQ, the analysis was carried out using the standard UEQ spreadsheet.

### AIMS Clinical Portal: SUS Results

All 26 participants completed the SUS questionnaire (100% response rate). The mean SUS score obtained was 82.88 (SD 13.07), with a median of 86.25. This score would be considered *good/excellent* according to the adjective rating scale developed by Bangor et al [41]. A breakdown of the participants’ answers to the SUS questions regarding the AIMS clinical portal is provided in Table 1.

**Table 1:** Participants’ answers to the System Usability Scale (SUS) questions for the Active and Independent Management System clinical portal (n=26).

Statements		Participants agreeing, n (%)	Participants disagreeing, n (%)	SUS scores, mean (SD)
**Positive statements**				
	I think that I would like to use this system frequently	23 (88)	2 (8)	4.00 (0.76)
	I thought the system was easy to use	24 (92)	1 (4)	4.50 (0.77)
	I found the various functions in this system were well integrated	22 (85)	2 (8)	4.50 (0.96)
	I would imagine that most people would learn to use this system very quickly	23 (88)	1 (4)	4.19 (0.65)
	I felt very confident using this system	24 (92)	1 (4)	4.38 (1.42)
**Negative statements**				
	I found the system unnecessarily complex	2 (8)	23 (88)	2.04 (0.73)
	I think that I would need the support of a technical person to be able to use this system	1 (4)	24 (92)	1.50 (0.77)
	I thought there was too much inconsistency in this system	0 (0)	23 (88)	1.62 (0.86)
	I found the system very cumbersome to use	3 (88)	21 (81)	1.73 (1.05)
	I needed to learn a lot of things before I could get going with this system	0 (0)	24 (92)	1.42 (0.65)

### AIMS Clinical Portal: UEQ Results

**Figure 3:**
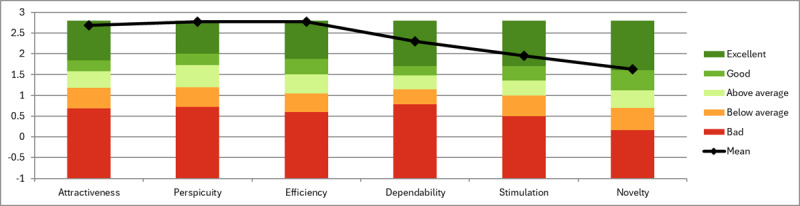
Bar chart of the Active and Independent Management System clinical portal User Experience Questionnaire results against the benchmarks.

**Figure 4:**
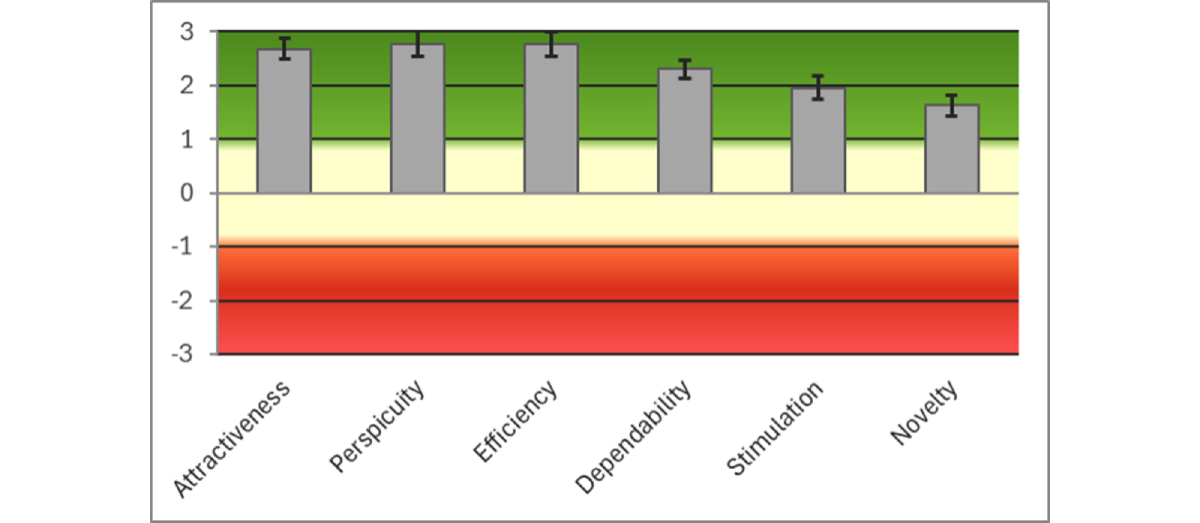
Bar chart of the Active and Independent Management System clinical portal data grouped into the 6 User Experience Questionnaire dimensions.

Invitations were sent to 12 (46%) of the 26 participants. Of these 12 participants, 10 (83%) completed the UEQ questionnaire. The means of the normalized scores (range −3 to +3) for the AIMS clinical portal were as follows: attractiveness=2.683 (SD 0.100), perspicuity=2.775 (SD 0.150), efficiency=2.775 (SD 0.130), dependability=2.300 (SD 0.080), stimulation=1.950 (SD 0.120), and novelty=1.625 (SD 0.090). Figure 3 shows the bar chart of the results for the AIMS clinical portal against the benchmarks, showing all dimensions classed as *excellent* and confirming the results from the SUS questionnaire. Table 2 provides the mean (SD) and variance of the normalized values for the items in the UEQ questionnaire for the AIMS clinical portal. In most cases, the values are very encouraging, with the exception of *conservative* and *innovative*, although this is still rated *good*. Figure 4 shows the bar chart of the data grouped into the 6 UEQ dimensions.

**Table 2:** Mean (SD) and variance of the normalized values for the items in the User Experience Questionnaire (UEQ) for the Active and Independent Management System clinical portal (n=10).

Scale	Left anchor of the scale	Right anchor of the scale	UEQ scores, mean (SD)	Variance
Attractiveness	Annoying	Enjoyable	1.9 (0.3)	0.1
Perspicuity	Not understandable	Understandable	2.6 (0.7)	0.5
Novelty	Creative	Dull	1.9 (0.3)	0.1
Perspicuity	Easy to learn	Difficult to learn	2.9 (0.3)	0.1
Stimulation	Valuable	Inferior	2.2 (0.6)	0.4
Stimulation	Boring	Exciting	1.5 (0.5)	0.3
Stimulation	Not interesting	Interesting	2.1 (0.6)	0.3
Dependability	Unpredictable	Predictable	1.8 (0.4)	0.2
Efficiency	Fast	Slow	2.8 (0.4)	0.2
Novelty	Inventive	Conventional	1.9 (0.3)	0.1
Dependability	Obstructive	Supportive	2.2 (0.6)	0.4
Attractiveness	Good	Bad	2.8 (0.4)	0.2
Perspicuity	Complicated	Easy	2.7 (0.5)	0.2
Attractiveness	Unlikable	Pleasing	2.7 (0.5)	0.2
Novelty	Usual	Leading edge	1.2 (0.4)	0.2
Attractiveness	Unpleasant	Pleasant	2.9 (0.3)	0.1
Dependability	Secure	Not secure	2.5 (0.5)	0.3
Stimulation	Motivating	Demotivating	2.0 (0.5)	0.2
Dependability	Meets expectations	Does not meet expectations	2.7 (0.5)	0.2
Efficiency	Inefficient	Efficient	2.8 (0.4)	0.2
Perspicuity	Clear	Confusing	2.9 (0.3)	0.1
Efficiency	Impractical	Practical	2.7 (0.5)	0.2
Efficiency	Organized	Cluttered	2.8 (0.4)	0.2
Attractiveness	Attractive	Unattractive	2.9 (0.3)	0.1
Attractiveness	Friendly	Unfriendly	2.9 (0.3)	0.1
Novelty	Conservative	Innovative	1.5 (0.5)	0.3

### AIMS Clinical Portal: Qualitative Feedback

To gain further insight into how users perceived the AIMS clinical portal, 3 additional questions were asked (refer to the following subsections).

#### Q1: What Do You Think Are the Advantages of This Portal?

Of the 26 participants, 20 (77%) answered this question. All clinicians (20/20, 100%) who answered the question thought that providing customized exercise videos after an operation was very useful, particularly for patients being able to use the system at home; 15 (75%) of the 20 clinicians also suggested that receiving immediate feedback on how patients were coping with the exercise regime was very helpful and meant that the regime could be easily customized for each patient based on how they were coping, which was a key advantage. In addition, 60% (12/20) of the clinicians considered ease of use an advantage. Example comments were as follows:

Really liked the exercise videos for the patients; they were professionally produced and highly relevant for rehabilitation.

Physiotherapist A

I’m pleased to see that patients automatically receive some feedback on how they are progressing with the rehabilitation exercises.

Physiotherapist B

#### Q2: What Do You Think Are the Disadvantages of This Portal?

Of the 26 participants, 12 (46%) answered this question. Of these 12 clinicians, 7 (58%) thought that the integration of the portal with the current IT systems may be a challenge, 3 (25%) thought that getting the staff to agree to use the portal may be a possible issue, and 2 (17%) thought that some staff members would need training on how to use it. Example comments were as follows:

One big issue that will have to be addressed at some point is integrating the software with hospital systems, as we ultimately need to have the patient progress data in their EHR [electronic health record].

Physiotherapist B

While the current system was intuitive and easy to use, I wonder whether some training will need to be provided when the features to add further videos and provide more customised feedback are added.

Physiotherapist C, an academic

#### Q3: Would You Change Anything?

Of the 26 participants, 17 (65%) answered this question. Of these 17 clinicians, 12 (71%) suggested that the ability to create more self-help advice for patients would be useful, and 5 (29%) suggested that having a larger data bank of exercise regimes would be helpful. Example comments were as follows:

It would be very helpful if more self-help could be added to the app to reduce the dependency on the volume of information sheets we provide to patients.

Physiotherapist D

The current set of videos are very relevant and of a high quality; however, it would be beneficial to be able to have a wider selection of videos to be able [to] select from.

Physiotherapist E

### AIMS App: SUS Results

The participants were selected during their first postoperative rehabilitation session (opportunistic recruitment). The recruitment of patients was carried out by a physiotherapist who would ask patients during their first session whether they were willing to participate in the study. The physiotherapist provided an information leaflet that explained what the study was about and how it could be used. All postoperative patients automatically qualified for the study; no one was excluded based on age, sex, or technical competency. The study did not collect any age- or sex-related information (a condition of the ethics approval for the study); therefore, it was not possible to provide information about patient demographics.

Of the 44 patients, 38 (86%) completed the SUS questionnaire. The mean SUS score obtained was 74.41 (SD 10.26), with a median of 77.50. This score would be considered *good* according to the adjective rating scale developed by Bangor et al [41]. A breakdown of the participants’ answers to the SUS questions for the AIMS app is provided in Table 3.

**Table 3:** Participants’ answers to the System Usability Scale (SUS) questions for the Active and Independent Management System app (n=38).

Statements		Participants agreeing, n (%)	Participants disagreeing, n (%)	SUS scores, mean (SD)
**Positive statements**				
	I think that I would like to use this system frequently	34 (89)	1 (3)	4.16 (0.68)
	I thought the system was easy to use	31 (82)	1 (3)	4.13 (0.62)
	I found the various functions in this system were well integrated	34 (89)	1 (3)	4.16 (0.68)
	I would imagine that most people would learn to use this system very quickly	32 (84)	2 (5)	3.92 (0.67)
	I felt very confident using this system	33 (87)	2 (5)	3.97 (0.68)
**Negative statements**				
	I found the system unnecessarily complex	1 (3)	32 (84)	2.00 (0.66)
	I think that I would need the support of a technical person to be able to use this system	1 (3)	32 (84)	1.92 (0.71)
	I thought there was too much inconsistency in this system	1 (3)	32 (84)	2.11 (0.56)
	I found the system very cumbersome to use	1 (3)	28 (74)	2.24 (0.59)
	I needed to learn a lot of things before I could get going with this system	2 (5)	26 (68)	2.32 (0.66)

### AIMS App: UEQ Results

**Figure 5:**
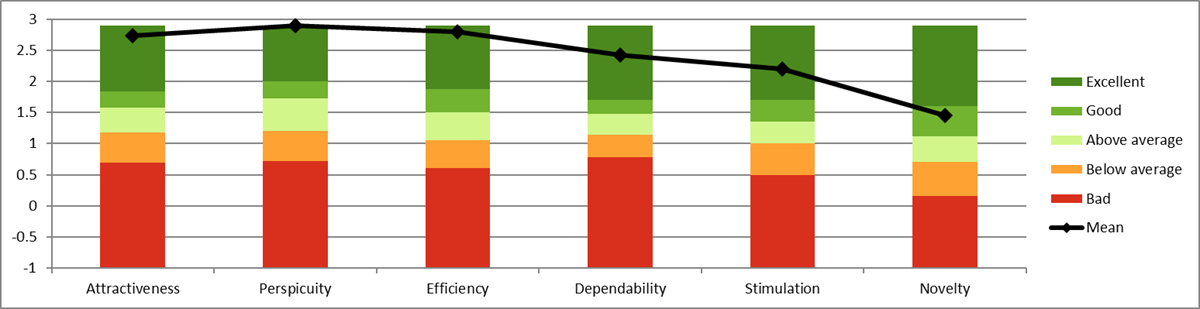
Bar chart of the Active and Independent Management System app User Experience Questionnaire results against the benchmarks.

**Figure 6:**
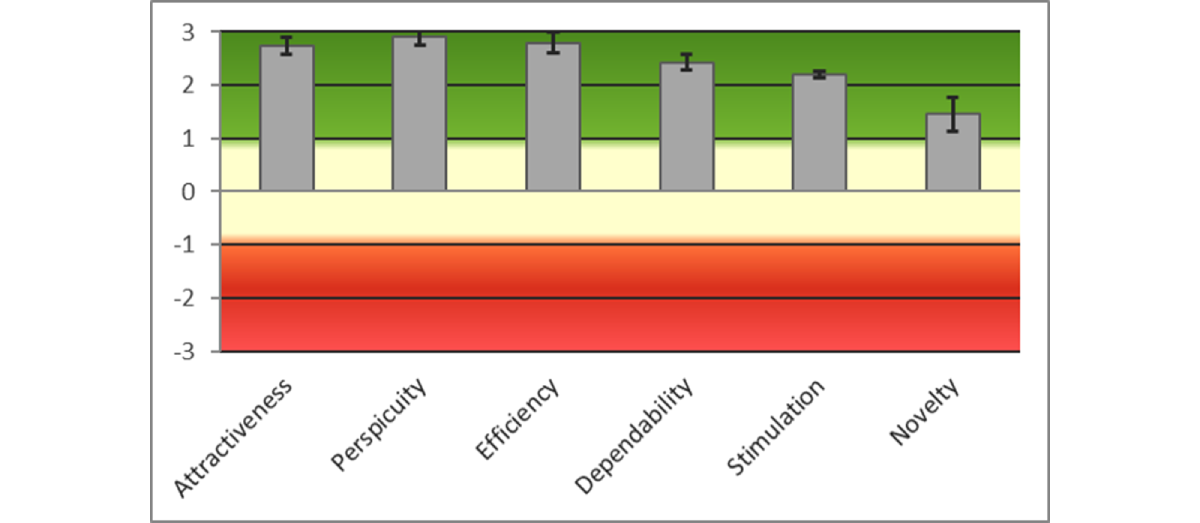
Bar chart of the Active and Independent Management System app data grouped into the 6 User Experience Questionnaire dimensions.

Invitations were sent to 12 (27%) of the 44 participants. Of these 12 patients, 10 (83%) completed the UEQ questionnaire. The means of the normalized scores (range −3 to +3) for the AIMS app were as follows: attractiveness=2.733 (SD 0.070), perspicuity=2.900 (SD 0.060), efficiency=2.800 (SD 0.090), dependability=2.425 (SD 0.060), stimulation=2.200 (SD 0.010), and novelty=1.450 (0.260). Figure 5 shows the bar chart of the results for the AIMS app against the benchmarks, with all dimensions classed as *excellent* (with the exception of *novelty*, which was classed as *good*), providing slightly better results than the SUS questionnaire. Table 4 gives the mean (SD) and variance of the normalized values for the items in the UEQ questionnaire for the AIMS app. In this case, all values are very encouraging. Figure 6 shows the bar chart for the data grouped into the 6 UEQ dimensions.

**Table 4:** Mean (SD) and variance of the normalized values for the items in the User Experience Questionnaire (UEQ) for the Active and Independent Management System app (n=10).

Scale	Left anchor of the scale	Right anchor of the scale	UEQ scores, mean (SD)	Variance
Attractiveness	Annoying	Enjoyable	1.9 (0.3)	0.1
Perspicuity	Not understandable	Understandable	2.6 (0.7)	0.5
Novelty	Creative	Dull	1.9 (0.3)	0.1
Perspicuity	Easy to learn	Difficult to learn	2.9 (0.3)	0.1
Stimulation	Valuable	Inferior	2.2 (0.6)	0.4
Stimulation	Boring	Exciting	1.5 (0.5)	0.3
Stimulation	Not interesting	Interesting	2.1 (0.6)	0.3
Dependability	Unpredictable	Predictable	1.8 (0.4)	0.2
Efficiency	Fast	Slow	2.8 (0.4)	0.2
Novelty	Inventive	Conventional	1.9 (0.3)	0.1
Dependability	Obstructive	Supportive	2.2 (0.6)	0.4
Attractiveness	Good	Bad	2.8 (0.4)	0.2
Perspicuity	Complicated	Easy	2.7 (0.5)	0.2
Attractiveness	Unlikable	Pleasing	2.7 (0.5)	0.2
Novelty	Usual	Leading edge	1.2 (0.4)	0.2
Attractiveness	Unpleasant	Pleasant	2.9 (0.3)	0.1
Dependability	Secure	Not secure	2.5 (0.5)	0.3
Stimulation	Motivating	Demotivating	2.0 (0.5)	0.2
Dependability	Meets expectations	Does not meet expectations	2.7 (0.5)	0.2
Efficiency	Inefficient	Efficient	2.8 (0.4)	0.2
Perspicuity	Clear	Confusing	2.9 (0.3)	0.1
Efficiency	Impractical	Practical	2.7 (0.5)	0.2
Efficiency	Organized	Cluttered	2.8 (0.4)	0.2
Attractiveness	Attractive	Unattractive	2.9 (0.3)	0.1
Attractiveness	Friendly	Unfriendly	2.9 (0.3)	0.1
Novelty	Conservative	Innovative	1.5 (0.5)	0.3

### AIMS App: Qualitative Feedback

To gain further insight into how users perceived the AIMS app, 3 additional questions were asked (refer to the following subsections).

#### Q1. What Do You Think Are the Advantages of This App?

Most of the participants (33/44, 75%) answered this question. Of the 33 participants, 26 (79%) thought that the exercise videos provided after an operation were very useful, 30 (91%) considered clinicians having immediate access to patient progress an advantage, and 24 (73%) considered ease of use an advantage. Example comments were as follows:

Having exercise videos that I can use both in the hospital and at home is a great help. While there is help on hand in the hospital if needed, being able to view the videos while at home is great.

Patient A

Loved the being able to access the videos on the tablet, was very helpful and the app was so easy to use.

Patient B

#### Q2. What Do You Think Are the Disadvantages of This App?

Only 8 (18%) of the 44 participants answered this question, and very few disadvantages were listed: 1 (13%) participant thought that the app could include some embedded videos for generic stretching exercises; 1 (13%) thought that the app might be too simple, and more functionality was required; and 6 (75%) thought that a self-help section would be beneficial. An example comment was as follows:

While the hospital provide[s] a number of leaflets on what to expect after the knee replacement, it would be handier of [sic] these were part of the app.

Patient C

#### Q3. Would You Change Anything?

Of the 44 participants, 17 (39%) answered this question. Of these 17 participants, 6 (35%) suggested more self-help, and 5 (29%) suggested having the ability to keep a daily or weekly diary of symptoms or pain. An example comment was as follows:

Would it be possible to have a section in the app to record how I am getting on with the videos and make notes on any symptoms I’m getting after the operation, particularly once I’m home?

Patient D

## Discussion

### Principal Findings

The main goal of this mixed methods study was to examine the usability of the AIMS platform from the perspectives of both patients and clinicians. Two well-known validated instruments were used to measure usability: the SUS and the UEQ. In all, 26 physiotherapists and health care professionals evaluated the AIMS clinical portal; and 44 patients in hospital for TKR, THR, or DHS evaluated the AIMS app. In terms of the RQs, the study has shown that both the AIMS clinical portal (RQ1) and the AIMS app (RQ2) have *good* to *excellent* usability scores, and this platform provides a solid foundation for the next phase of research, which will involve evaluating its effectiveness in improving patient outcomes after TKR, THR, or DHS. In addition, useful qualitative information was obtained from participants through a set of open-ended questions.

On the basis of the literature reviewed in the Re-Enablement DHIs subsection, it seems that smartphones and the web are the 2 main platforms used to provide re-enablement DHIs after TKA or THA. The platforms have been identified to be used by patients who will receive the instructions in the form of video, text, and interactive game as well as by clinicians who can create custom treatment plans for patients. The AIMS platform provides similar functionality to the systems found in the literature, with a web-based clinical portal and a mobile app for patients. The AIMS platform mainly presents content in video and text, which is similar to the majority of the systems discussed in the Re-Enablement DHIs subsection. Text and video are considered to be effective in presenting rehabilitation content to patients because they allow a wider level of proficiency in information and communications technology. Compared to static images, we considered videos to be more engaging, although further research should be conducted to investigate this. Some studies, such as those by Hussain et al [23], van Dijk-Huisman et al [25], and Bell et al [27], used sensors from wearable devices and mobile phones, while Bäcker et al [28] developed their own custom sensor. Personalization features that allow the system to customize activities for patients were only evident in the studies by Hussain et al [23], van Dijk-Huisman et al [25], and Bell et al [27]. Currently, the AIMS platform does not use sensors or have any personalization features, but these will be considered for the next phase of the research. A summary comparing the literature reviewed with our study can be found in Multimedia Appendix 1 [23-30].

In terms of limitations, to overcome major privacy concerns, a condition of the ethics approval for the study was that all data had to be anonymized; therefore, neither could we perform a demographic analysis nor conduct follow-up monitoring of the progress of a more informed patient after they left the hospital. As this study’s main focus was on usability, the recruitment of participating patients was carried out during rehabilitation sessions. This method did not allow us to conduct a randomized study, and there were no control and experimental groups. Furthermore, due to ethics approval restrictions, we were not able to directly observe the experiment and had to use questionnaires and interviews conducted by the clinicians during the rehabilitation sessions. The experiment did not use any additional sensing technologies to monitor user progress and relied on the patient’s input and feedback. Future studies will aim to overcome these limitations.

Since this study was carried out, the platform has been improved to include additional support videos for patients, ideas for which emerged from the qualitative feedback, and a second usability study is underway to ensure that results are consistent with this initial study (Multimedia Appendix 1).

### Conclusions

This study aimed to assess the usability of a re-enablement platform called AIMS, designed to address the *de-enablement* often experienced by hospitalized older adults most at risk. Usability was measured using 2 common validated instruments: the 10-item SUS and, for more detailed analysis, the 26-item UEQ. The AIMS clinical portal was evaluated by 26 physiotherapists and health care professionals; and 44 patients undergoing TKR, THR, or DHS assessed the AIMS app. Overall, both the AIMS clinical portal and the AIMS app received *good* to *excellent* usability scores, providing a solid foundation for future research on their effectiveness in improving patient outcomes after joint replacements. Optimal rehabilitation programs for orthopedic joint replacement patients can lead to a quicker return to normal function, faster hospital discharge, and higher patient satisfaction.

## Appendix

Multimedia Appendix 1 Comparison with the literature reviewed.

## Abbreviations

AIMS: Active and Independent Management System
DHI: digital health intervention
DHS: dynamic hip screw
IC: integrated care
mHealth: mobile health
POD1: postoperative day 1
RQ: research question
SUS: System Usability Scale
THA: total hip arthroplasty
THR: total hip replacement
TKA: total knee arthroplasty
TKR: total knee replacement
UEQ: User Experience Questionnaire
